# The Pupal Pigmentation Pattern and Pupal Development in the Species of *Aphytis* Howard (Hymenoptera: Aphelinidae)

**DOI:** 10.3390/insects12050399

**Published:** 2021-04-30

**Authors:** Zhuhong Wang, Yu Si, Hui Zhang, Zhengli Zhang, Andrew Polaszek, Jian Huang

**Affiliations:** 1 State Key Laboratory of Ecological Pest Control for Fujian and Taiwan Crops, Fujian Agriculture and Forestry University, Fuzhou 350002, China; wzhuhong@126.com (Z.W.); siyuxsh@sina.com (Y.S.); 2170203003@fafu.edu.cn (H.Z.); zhenglizzl@126.com (Z.Z.); 2 Department of Life Sciences, Natural History Museum, Cromwell Road, London SW7 5BD, UK

**Keywords:** *Aphytis*, pupal coloration, biocontrol agents

## Abstract

**Simple Summary:**

*Aphytis* species (Hymenoptera: Aphelinidae) have been considered as the most important natural enemies in biological control of armoured scale insects (Hemiptera: Diaspididae). However, it is difficult to identify the species of the genus, particularly that of the *A. lingnanensis* group, based on adult morphological characters. Although the differences in pupal pigmentation of *Aphytis* species have been documented previously, they have not been much used as taxonomic characters for *Aphytis* species identification. In this study, we present four characteristic pigmentation patterns of *Aphytis* pupae, together with photographs, including the categories of: entirely yellow, partly dark brown, entirely or predominantly black, and partly black pupae. No significant intra-specific variation in pupal colour pattern was detected despite relatively high numbers of specimens examined, many from multiple, and different, origins. We summarize the present status of pupal pigmentation in the described species of *Aphytis*, according to the species groups, which could be used as an important supplementary diagnostic character for distinguishing species of *Aphytis*, especially closely-related species.

**Abstract:**

Species identification of *Aphytis* on the basis of adult morphology is extremely difficult, especially in the *A. lingnanensis* group, with several cryptic species. Pupal pigmentation could be used as one of the taxonomic characters for *Aphytis* species, and in some instances, pupal pigmentation actually provided the first clue to the distinctness of cryptic *Aphytis* species. The present study investigated the full-grown larvae or younger pupae of *Aphytis* species, and pupal pigmentation and pupal development were observed and photographed. Four characteristic pigmentation patterns of *Aphytis* pupae were summarized including: entirely yellow, partly dark brown, entirely or predominantly black, and partly black. The species in the *chilensis* and *mytilaspidis* groups, and some unassigned species, generally have entirely, or predominantly and or partly black pupae. The species in the *chrysomphali*, *funicularis*, and *proclia* groups generally have the pupae entirely yellow. The species of the *lingnanensis* group have the pupae both entirely yellow, e.g., *A. fisheri*, and partly dark brown pupae, e.g., *A. lingnanensis*, *A. holoxanthus* and *A. melinus*. The pupae of *Aphytis* species in this study had a developmental duration of about 5–8 days at 27 ± 1 °C, 70 ± 5% RH and a photoperiod of 10L: 14D. It was found that the pupal skin was always melanized at the beginning stage, generally in the first day, among the pigmented pupae of *Aphytis* species. As development continued, the pigmentation became darker and the eye colour changed from pale red/brown to green. No significant intra-specific variation in pupal colour pattern was detected despite relatively high numbers of specimens examined, many from multiple, and different, origins. Overall, our study indicates that pupal pigmentation could be more helpful in species identification of *Aphytis*.

## 1. Introduction

The species of *Aphytis* Howard (Hymenoptera: Aphelinidae) are among the most important ectoparasitoids of armoured scale insects (Hemiptera: Diaspididae), and many of them were utilized successfully in biological control projects of important armoured scale pests around the world. For instance, *A. lingnanensis* Compere from Guangdong, China in 1947 and *A. melinus* DeBach from India and Pakistan in 1956–1957 were introduced into California, USA against the California red scale, *Aonidiella aurantii* (Maskell); *A. holoxanthus* DeBach was introduced into Israel in 1956–1957 from Hong Kong, China against the Florida red scale, *Chrysomphalus aonidum* (L.), and *A. yanonensis* DeBach & Rosen was introduced into Japan in 1980 from Sichuan, China against the arrowhead scale, *Unaspis yanonensis* (Kuwana) [1,2,3].

However, species identification of *Aphytis* is extremely difficult on the basis of adult morphology, especially in the *A. lingnanensis* group, with several cryptic species. This difficulty is due to their minute size (rarely exceeding one millimeter in length), the propodeal crenulae not easy to observe, and the relative scarcity of reliable distinguishing characters, as well as often unsatisfactory slide-mounted specimens without clearly visible characters [1,4].

Taylor [5] was the first to notice differences in pupal pigmentation, the yellow pupae and the dark pupae, between the closely related species *A. chrysomphali* (Mercet) and what was probably *A. holoxanthus*. Pupal pigmentation was consequently used as one of the taxonomic characters for *Aphytis* species not readily separable using adult morphology [1,6,7,8,9,10,11,12]. In some instances, pupal pigmentation actually provided the first clue to the distinctness of cryptic *Aphytis* species. *A. lingnanensis*, for example, was first recognized as distinct from *A. chrysomphali* on the basis of this character [7]. In the *A. lingnanensis* group, *A. fisheri* DeBach, *A. holoxanthus* and *A. melinus* are considered sibling or near-sibling species, almost inseparable at the adult stage, but they are different in pupal pigmentation. The pupae of *A. holoxanthus* exhibit dark brown pigmentation on both the mesosomal sterna and the base of the metasomal sterna, those of *A. melinus* are pigmented only on the mesosomal sterna, whereas the pupae of *A. fisheri* are entirely yellow [1]. Yasnosh [13] provided a key for identification of the *Aphytis* species from the pupae and meconia (in the former USSR), and Prinsloo [14] gave a key to distinguishing the pupae of *Aphytis* parasitic on the California red scale on citrus in South Africa.

In southern China there is a major *Aphytis* parasitoid associated with the Florida red scale on citrus, which was mistakenly identified as *A. chrysomphali* for long time, and later was clarified as *A. holoxanthus* on the basis of the pupal pigmentation (the former with yellow pupa and the latter with partly dark brown pupa), as well as the characters of propodeal crenulae (the former belonging to *A. chrysomphali* group and the latter to *A. lingnanensis* group) and other adult characters [3,15].

Therefore, the aim of this study is to observe and document the taxonomic characters of pupal pigmentation and pupal development, in order to provide information useful for the separation of *Aphytis* species. We are not aware of any between-species or species group diagnostic studies in other Aphelinidae, nor indeed any other Hymenoptera genera.

## 2. Materials and Methods

### 2.1. Culture of Aphytis Pupae

Several species of armoured scale insects were collected from citrus and ornamental plants in the fields. The full-grown larvae or younger pupae of *Aphytis* species were removed individually from parasitized scale insect hosts, and placed each in an individually numbered plastic box (3 cm in diameter, 1.5 cm high), in which a filter paper was placed. The boxes were put into a climatic chamber for pupa culture, under the controlled conditions at 27± 1 °C, 70 ± 5% RH and a photoperiod of 10L: 14D. The pupal pigmentation and pupal development were observed and photographed daily, also adding a drop of water on the filter paper to maintain humidity. Pigmentation of mature, green-eyed pupae was recorded and compared for *Aphytis* species. Length of pupal development was measured from the time of larval pupation until the formation of the mature pupa, indicated by the change in eye pigment to green.

### 2.2. Identification of Aphytis Species

The body colour of *Aphytis* species after emergence was initially photographed, and the specimens were preserved in 100% ethanol. Later the adult *Aphytis* specimens were photographed for details of the propodeal crenulae and thoracic tergum, and slide-mounted for species identification following the method outlined by Noyes [4].

### 2.3. Photography of Specimens

Specimens were photographed with a DS-Ri2 camera (Nikon, Tokyo, Japan) attached to a Nikon SMZ18 microscope with NIS-Elements D software and a DSC-T900 camera (Sony, Tokyo, Japan). The slide-mounted specimens were photographed using the Nikon DS-Ri2 camera and the same software attached to a Nikon Ni microscope equipped with differential interference contrast.

## 3. Results

Four characteristic pigmentation patterns of *Aphytis* pupae were recorded in this study, including: 1. entirely yellow; 2. partly dark brown; 3. entirely or predominantly black, and 4. partly black.

### 3.1. Type 1, Entirely Yellow Pupa

In this category, four *Aphytis* species with entirely yellow pupae were from the *funicularis*, *chrysomphali*, *lingnanensis* and *proclia* groups.

#### 3.1.1. *Aphytis gordoni* DeBach & Rosen (*funicularis* Group)

**Figure 1:**
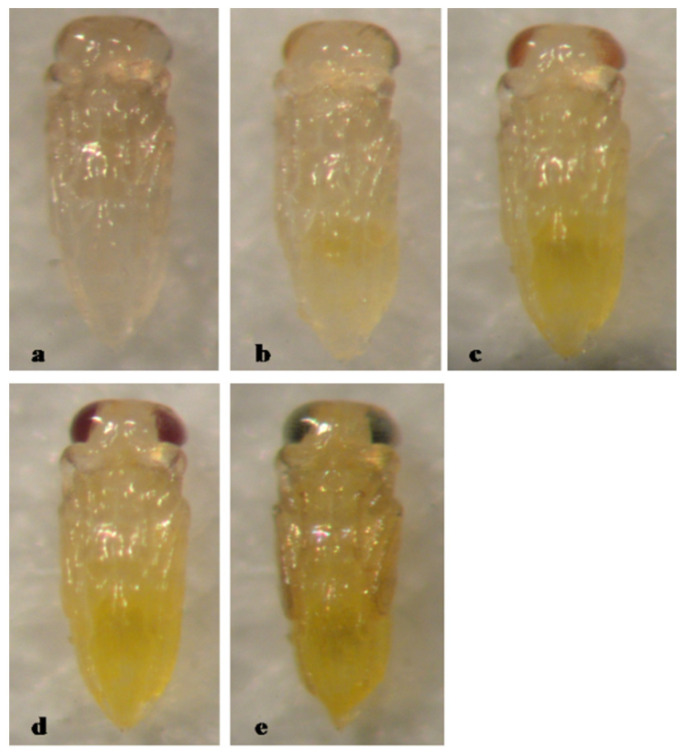
*Aphytis gordoni* DeBach & Rosen. ♀ pupa, ventral view: (**a**) 1-day old, (**b**) 2-day old, (**c**) 3-day old, (**d**) 4-day old, and (**e**) 5-day old (mature).

The pupa of *A. gordoni* is entirely yellow, with pupal development lasting 5 days (Figure 1).

Material examined. 1♀, ex *Pinnaspis theae* (Maskell) ♂ on tea. China: Fujian, Fuzhou, Jinshan, Fujian Agriculture and Forestry University (hereafter FAFU), tea plantation, 10.vi.2015, coll. Zhongchun Wen; 5♀, ex *Pinnaspis theae* ♂ on tea. China: Fujian, Fuzhou, Jinshan, FAFU, tea plantation, 13–28.ix.2015, coll. Taishan Zhou; 1♀, ex an unidentified diaspidid ♂ on bamboo, China: Fujian, Fuzhou, Jinshan, FAFU, 2.xi.2015, coll. Zhengli Zhang and Hui Zhang; 2♀2♂, ex an unidentified diaspidid ♂ on *Litchi chinensis* Sonn. China: Fujian, Fuzhou, Jinshan, FAFU, 14.vi.2015, coll. Zhongchun Wen; 2♀2♂, ex *Aulacaspis yabunikkei* Kuwana ♂ on *Cinnamomum camphora* (L.) Presl. China: Fujian, Zhangzhou, 24.iv.2015, coll. Zhengli Zhang.

#### 3.1.2. *Aphytis lepidosaphes* Compere (*chrysomphali* Group)

**Figure 2:**
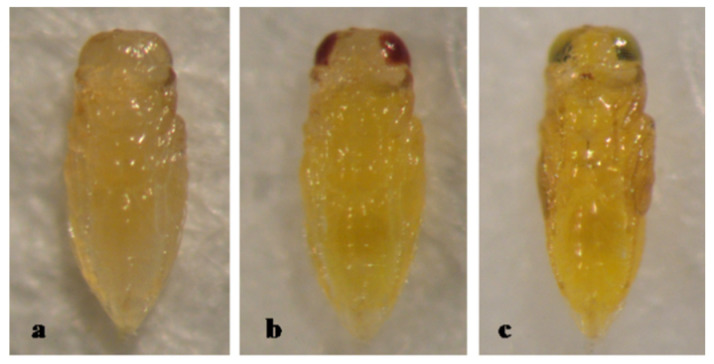
*Aphytis lepidosaphes* Compere. ♀ pupa, ventral view: (**a**) 1-day old, (**b**) 4-day old, and (**c**) 7-day old (mature).

The pupa of *A. lepidosaphes* is entirely yellow, with pupal development lasting 7 days (Figure 2).

Material examined. 4♀, ex *Cornuaspis beckii* (Newman) on *Murraya exotica* L. China: Fujian, Fuzhou, Jinshan, FAFU, 14.xii.2014, coll. Xiurong Peng; 6♀, ex *Cornuaspis beckii* (Newman) on *Ficus microcarpa* L., China: Fujian, Fuzhou, Jinshan, FAFU, 15.xii.2014, coll. Zhengli Zhang.

#### 3.1.3. *Aphytis fisheri* DeBach (*lingnanensis* Group)

**Figure 3:**
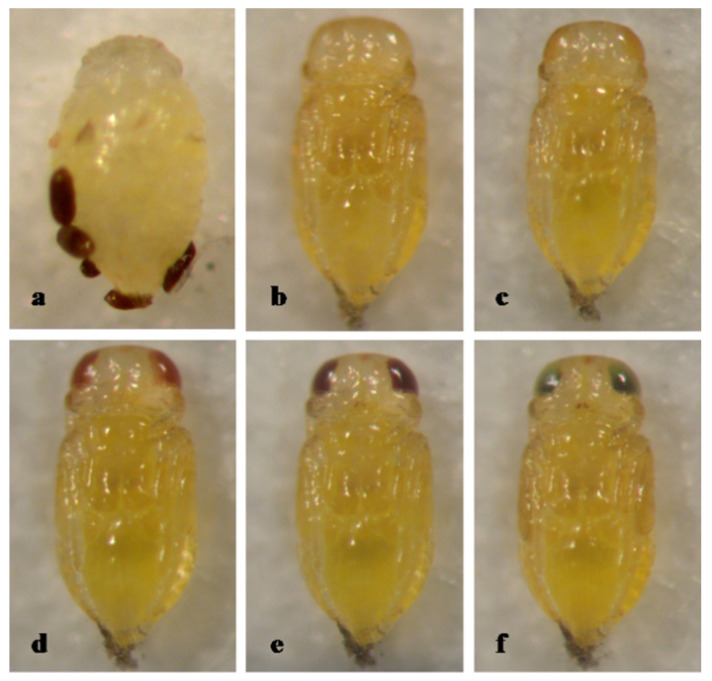
*Aphytis fisheri* DeBach. (**a**) a prepupa and meconia, ♀ pupa, ventral view: (**b**) 1-day old, (**c**) 2-day old, (**d**) 3-day old, (**e**) 4-day old, and (**f**) 5-day old (mature).

The pupa of *A. fisheri* is entirely yellow, with pupal development lasting 5 days (Figure 3).

Material examined. 2♀2♂, ex an unidentified diaspidid on *Dianella ensifolia* (L.). China: Fujian, Fuzhou, Jinshan, FAFU, 17.iv., 5.v.2015, coll. Yu Si and Zhongchun Wen; 1♀, ex *Aonidiella citrina* (Coquillett) on citrus. China: Fujian, Jian’ou, 4.xi.2015, coll. Xu Cai; 2♀2♂, ex an unidentified diaspidid on grass, China: Fujian, Fuzhou, 11.vi.2015, coll. Yu Si; 1♀, ex an unidentified diaspidid on *Ficus microcarpa* L. China: Fujian, Fuzhou, Jinshan, FAFU, 15.xii.2014, coll. Zhengli Zhang; 1♀, ex *Pseudaulacaspis cockerelli* (Cooley) on *Michelia alba* DC. China: Fujian, Xiamen, 31.i.2015, coll. Jian Huang and Hui Zhang.

#### 3.1.4. *Aphytis hispanicus* (Mercet) (*proclia* Group)

**Figure 4:**
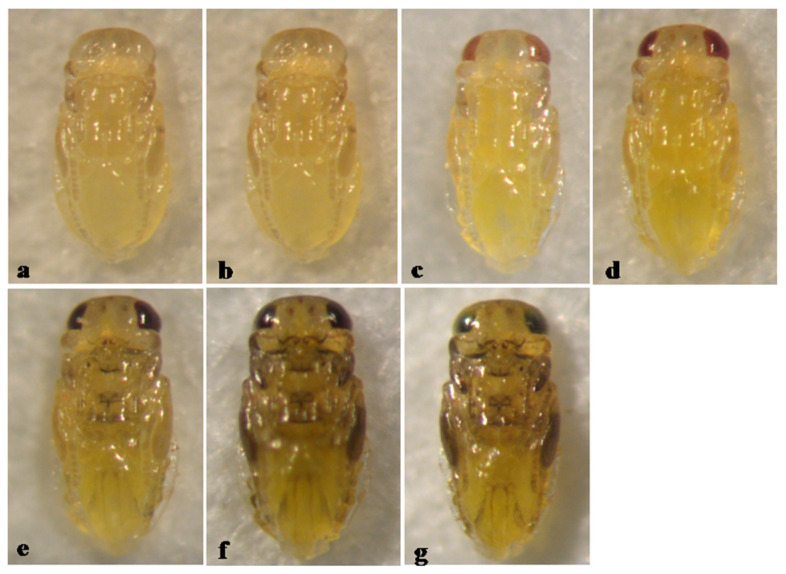
*Aphytis hispanicus* (Mercet). ♀ pupa, ventral view: (**a**) 1-day old, (**b**) 2-day old, (**c**) 3-day old, (**d**) 4-day old, (**e**) 5-day old, (**f**) 6-day old, and (**g**) 7-day old (mature).

The pupa of *A. hispanicus* is actually entirely yellow. The melanization seen in the late stage of pupal development (Figure 4e–g) is that of the developing adult, evident through the integument of the fully developed pupa. The pupal development duration is 7 days (Figure 4).

Material examined. 4♀, ex an unidentified diaspidid on *Ilex chinensis* Sims. China: Fujian, Xiamen, 31.i.2015, coll. Jian Huang and Hui Zhang; 4♀2♂, ex *Pseudaulacaspis cockerelli* (Cooley) on *Michelia figo* (Lour.) Spreng. China: Fujian, Fuzhou, Jinshan, FAFU, 10.ii.2015, Xiurong Peng.

### 3.2. Type 2, Partly Dark Brown Pupa

In this type, the *Aphytis* species observed were characterized by dark brown pigmentation on both the thoracic and abdominal sterna, or on the thoracic and basal abdominal sterna, or only on the thoracic sterna of the pupae.

#### 3.2.1. *Aphytis lingnanensis* Compere (*lingnanensis* Group)

**Figure 5:**
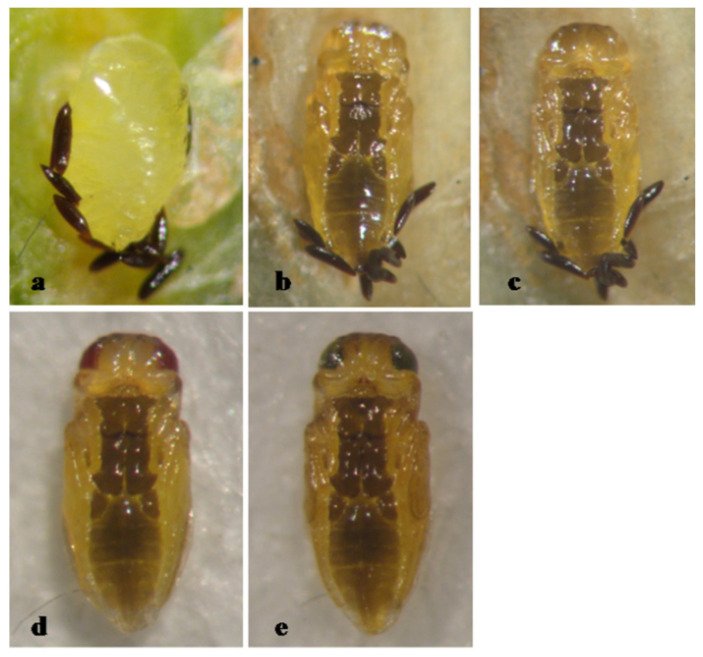
*Aphytis lingnanensis* Compere. (**a**) prepupa and meconia, ♀ pupa, ventral view: (**b**) 1-day old, (**c**) 2-day old, (**d**) 4-day old, and (**e**) 6-day old (mature).

*A. lingnanensis* is characterized by dark brown pigmentation on both the thoracic and abdominal sterna of the pupa, with pupal development lasting 6 days (Figure 5).

Material examined. 2♀, ex *Aonidiella orientalis* (Newstead) on *Cycas revoluta* Thunb. China: Fujian, Fuzhou, Jinshan, FAFU, 15, 23.xii.2014, coll. Zhengli Zhang; 2♀1♂, ex *Pseudaonidia trilobitiformis* (Green) on *Ficus microcarpa* L. cv. Golden Leaves, China: Fujian, Fuzhou, Jinshan, FAFU, 19.vi.2015, coll. Yu Si and Zhongchun Wen; 1♂, ex *Pinnaspis theae* on tea, China: Fujian, Fuzhou, Jinshan, FAFU, 10.vi.2015, coll. Zhongchun Wen; 1♂, ex *Pseudaulacaspis cockerelli* (Cooley) on *Michelia figo* (Lour.) Spreng. China: Fujian, Fuzhou, 19.vi.2015, coll. Yu Si; 2♀1♂, ex *Chrysomphalus aonidum* (L.) on banana, China: Fujian, Fuzhou, Jinshan, FAFU, 22.iv.2015, coll. Yu Si; 1♂, ex *Chrysomphalus aonidum* (L.) on *Ficus microcarpa* L. China: Fujian, Fuzhou, Jinshan, FAFU, 10.xi.2014, coll. Xiurong Peng.

#### 3.2.2. *Aphytis holoxanthus* DeBach (*lingnanensis* Group)

**Figure 6:**
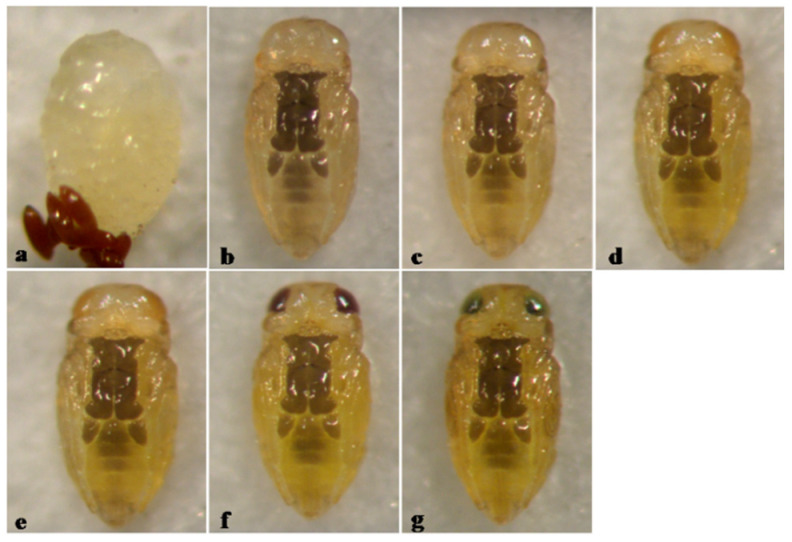
*Aphytis holoxanthus* DeBach. (**a**) prepupa and meconia, ♀ pupa, ventral view: (**b**) 1-day old, (**c**) 2-day old, (**d**) 3-day old, (**e**) 4-day old, (**f**) 5-day old, and (**g**) 6-day old (mature).

*A. holoxanthus* is characterized by dark brown pigmentationon on the thoracic and basal abdominal sterna of the pupa, with pupal development lasting 6 days (Figure 6).

Material examined. 11♀3♂, ex *Chrysomphalus aonidum* (L.) on *Citrus maxima* (Burm.) Merr. China: Fujian, Fuzhou, Pudang, 27.iii.2015, coll. Jian Huang and Zhengli Zhang; 2♀, ex *Pseudaulacaspis cockerelli* (Cooley) on *Michelia figo* (Lour.) Spreng. China: Fujian, Fuzhou, 10.vi.2015, coll. Zhongchun Wen; 2♀2♂, ex *Chrysomphalus aonidum* (L.) on *Osmanthus fragrans* (Thunb.) Lour. China: Fujian, Fuzhou, Jinshan, FAFU, 10.v.2015, coll. Zhengli Zhang; 1♀2♂, ex *Chrysomphalus aonidum* (L.) on citrus, China: Fujian, Xiamen, Tong’an, 23.iv.2015, coll. Jian Huang, Hui Zhang and Zhengli Zhang; 5♀3♂, ex an unidentified diaspidid on *Nerium indicum* Mill. China: Fujian, Fuzhou, 10.ix.2015, coll. Yu Si and Zhongchun Wen.

#### 3.2.3. *Aphytis melinus* DeBach (*lingnanensis* Group)

**Figure 7:**
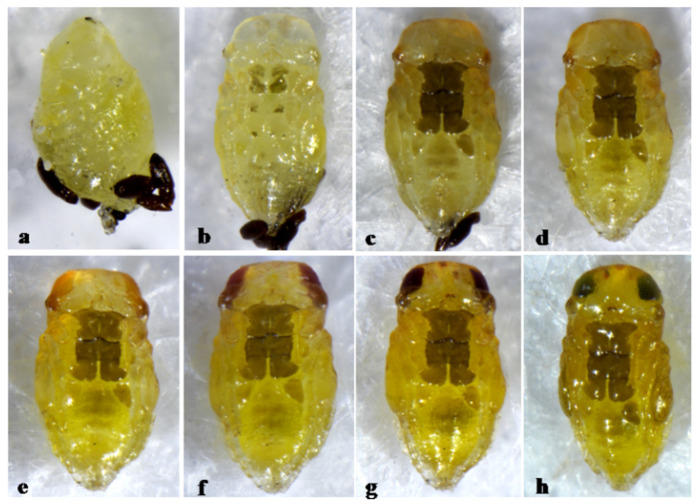
*Aphytis melinus* DeBach. (**a**) prepupa and meconia. ♀ pupa, ventral view: (**b**) 1-day old, (**c**) 2-day old, (**d**) 3-day old, (**e**) 4-day old, (**f**) 5-day old, (**g**) 6-day old, and (**h**) 7-day old (mature).

*A. melinus* is characterized by dark brown pigmentation only on the thoracic sterna of the pupa, with pupal development lasting 6 days (Figure 7).

Material examined. 1♀, ex *Chrysomphalus aonidum* (L.) on *Ficus microcarpa*. China: Fujian, Fuzhou, Jinshan, FAFU, 16.i.2018, coll. Junhui Zhou; 4♀, ex an unidentified diaspidid on *Dianella ensifolia* (L.) DC. China: Fujian, Fuzhou, Jinshan, FAFU, 4.xii.2015, coll. Zhongchun Wen; 1♂, ex an unidentified diaspidid on *Ficus microcarpa* L. China: Fujian, Fuzhou, Jinshan, FAFU, 23.xii.2014, coll. Zhengli Zhang; 1♀, ex *Chrysomphalus aonidum* (L.) on *Ficus microcarpa* L. China: Fujian, Fuzhou, 26.xii.2017, coll. Yu Si; 1♀, ex an unidentified diaspidid on citrus, China: Fujian, Jian’ou, 4.xi.2015, coll. Xu Cai; 1♀, ex *Chrysomphalus aonidum* (L.) on citrus, China: Fujian, Fuzhou, Minhou, 3.i.2018, coll. Yu Si and Junhui Zhou; 1♀1♂, ex *Chrysomphalus aonidum* (L.) on *Murraya exotica* L. China: Fujian, Fuzhou, Jinshan, FAFU, 4.ii.2015, coll. Xiurong Peng; 1♂, ex *Chrysomphalus aonidum* (L.) on *Ficus microcarpa* L. China: Fujian, Fuzhou, Jinshan, FAFU, 19.vi.2015, coll. Yu Si and Zhongchun Wen.

### 3.3. Type 3, Entirely or Predominantly Black Pupa

#### 3.3.1. *Aphytis longicaudus* Rosen & DeBach (Unassigned Species)

**Figure 8:**
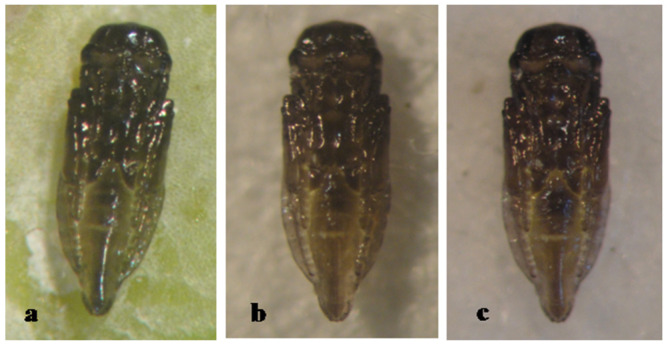
*Aphytis longicaudus* Rosen & DeBach. ♀ pupa, ventral view: (**a**) 1-day old, (**b**) 2-day old, and (**c**) 8-day (mature).

The pupa of *A. longicaudus* is entirely black, with pupal development lasting 8 days (Figure 8).

Material examined. 1♀1♂, ex *Pseudaonidia trilobitiformis* (Green) on *Psidium guajava* L. China: Fujian, Fuzhou, Jinshan, FAFU, 3, 23.i.2015, coll. Zhengli Zhang; 3♀, ex *Pseudaonidia trilobitiformis* (Green) on *Citrus maxima* (Burm) Merr. China: Yunnan, Jinghong, 22.xi.2017, coll. Jian Huang and Zhuhong Wang; 4♀, ex *Pseudaonidia trilobitiformis* (Green) on tree, China: Fujian, Jiangle, ix.2011, Lianbin Ye.

#### 3.3.2. *Aphytis sankarani* Rosen & DeBach (Species Group Placement Intermediate between *lingnanensis* and *mytilaspidis* Groups)

**Figure 9:**
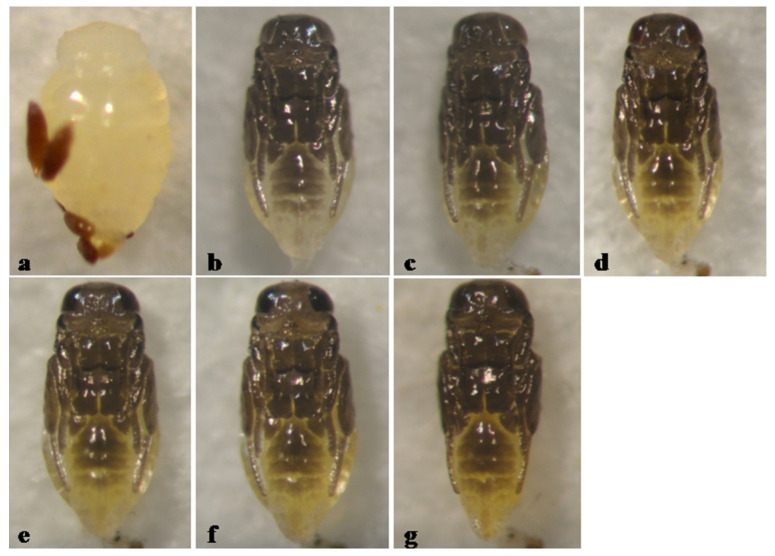
*Aphytis sankarani* Rosen & DeBach. (**a**) prepupa and meconia, ♀ pupa, ventral view: (**b**) 1-day old, (**c**) 2-day old, (**d**) 3-day old, (**e**) 4-day old, (**f**) 5-day old, and (**g**) 6-day old (mature).

The pupa of *A. sankarani* is predominantly black, except for being pale on the distad abdominal sterna, with pupal development lasting 6 days (Figure 9).

Material examined. 2♀, ex *Pseudaulacaspis cockerelli* (Cooley) on *Excoecaria cochinchinensis* Lour. China: Fujian, Fuzhou, Jinshan, FAFU, 10.ii.2015, coll. Hui Zhang; 3♀1♂, ex *Pseudaulacaspis cockerelli* (Cooley) on *Michelia figo* (Lour.) Spreng. China: Fujian, Fuzhou, Jinshan, FAFU, 14.xii.2014, coll. Xiurong Peng; 10♀6♂, ex *Pseudaulacaspis cockerelli* (Cooley) on *Michelia figo* (Lour.) Spreng. China: Fujian, Fuzhou, Jinshan, FAFU, 6.iv.2015, coll. Yu Si and Zhongchun Wen; 4♀4♂, ex *Pseudaulacaspis cockerelli* (Cooley) on *Michelia figo* (Lour.) Spreng. China: Fujian, Fuzhou, Jinshan, FAFU, 16.vi.2015, coll. Zhongchun Wen.

### 3.4. Type 4, Partly Black Pupa

#### *Aphytis* sp. (Unassigned Species)

**Figure 10:**
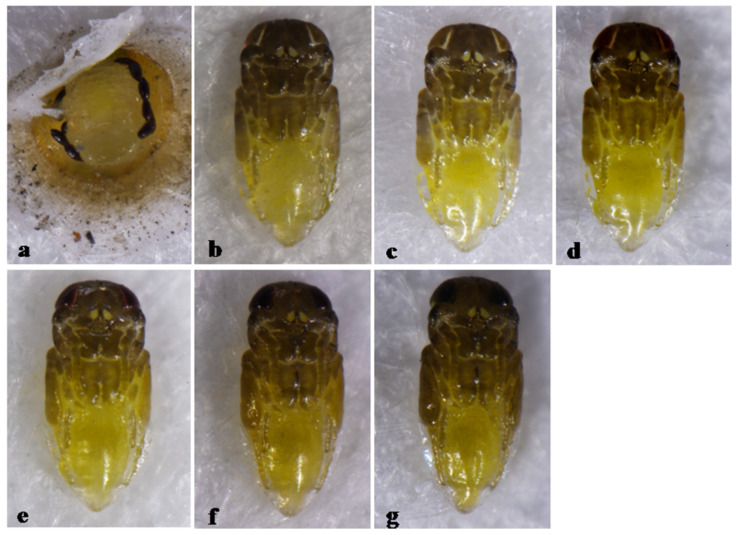
*Aphytis* sp. (**a**) prepupa and meconia on the scale, ♀ pupa, ventral view: (**b**) 1-day old, (**c**)2-day old, (**d**) 3-day old, (**e**) 4-day old, (**f**) 5-day old, and (**g**) 6-day old (mature).

The pupa of *Aphytis* sp. is partly black except for yellow on the abdominal sterna, with pupal development lasting 6 days (Figure 10).

Material examined. 3♀, ex an unidentified diaspidid on *Citrus maxima* (Burm.) Merr. China: Fujian, Fuzhou, Jinshan, FAFU, 10.xii.2017, coll. Yu Si and Junhui Zhou; 5♀3♂, ex *Parlatoria zizyphus* (Lucas) on citrus, China: Fujian, Fuzhou, Jinshan, FAFU, 9.vii.2018, coll. Yu Si and Junhui Zhou.

### 3.5. Pupal Development

The pupae of *Aphytis* species in this study had a developmental duration of about 5–8 days at 27 ± 1 °C, 70 ± 5% RH and a photoperiod of 10L: 14D. The experiments showed that the pupal skin was always melanized at beginning stage, generally in the first day, among the pigmented pupae of *Aphytis* species. As development continued, the pigmentation became darker and the eye colour from pale red/brown to green/black brown.

## 4. Discussion

Until now less than one-third of the known species of *Aphytis* have had their pupal pigmentation described (Table 1).

Of the known species of *Aphytis* (Table 1), the species in the *chilensis* and *mytilaspidis* groups, and some unassigned species, generally have entirely, or predominantly and or partly black pupae.

The species in the *chrysomphali*, *funicularis*, and *proclia* groups generally have the pupae entirely yellow. However, the pupal pigmentation of the *proclia* group seems to be generally mottled with fuscous; wing pads and appendages appear fuscous to black; dark areas or furcae are visible on both dorsal and ventral surfaces of mesosoma and metasoma; metasomal segments with dark patches dorso-laterally. Actually the pupal skin itself is usually not melanized at all. It is straw-yellow, and the melanization seen is that of the developing adult. Coarse, black spines are also usually evident through the integument of the fully developed pupa [1,8,9]. By comparison, the pupal skins of other types are melanized.

The species of the *lingnanensis* group have the pupae entirely yellow, e.g., *A. fisheri*, and partly dark brown pupae, e.g., *A. lingnanensis*, *A. holoxanthus* and *A. melinus*, which are almost inseparable by adult characters, but their differences in pupal pigmentation provides a method to separate these species.

## 5. Conclusions

This study summarized four characteristic pigmentation patterns of *Aphytis* pupae, including entirely yellow, partly dark brown, entirely or predominantly black, and partly black. No significant intra-specific variation in pupal colour pattern was detected despite relatively high numbers of specimens examined, many from multiple, and different, origins. Although one type of pupal pigmentation is shared with more than one species group, and pupal pigmentation is somewhat variable at times, for example the new pigmentation patterns were reported in some individuals of *A. melinus* and *A. chrysomphali* species respectively [33], the pupal pigmentation certainly may be regarded as an important supplementary diagnostic character, based on the morphological characters, and may even serve as a convenient shortcut to the separation of certain closely related species of *Aphytis*.

## Figures and Tables

**Table 1:** The present status of pupal pigmentation described in the known species of *Aphytis* ^(1)^.

Sp. Group	No. of sp. + Related sp.	No. of sp. Recorded with Pupal Pigmentation	Type of Pupal Pigmentation	References
*chilensis*	5 + 2	2	entirely or partly black	[1,16]
*chrysomphali*	17	5	entirely yellow, or some with brownish appendages	[1,3,16,17,18,19]
*funicularis*	5	2	entirely yellow	[1,20]
*lingnanensis*	19	11	partly dark brown; entirely yellow	[1,3,9,17,19,21,22,23,24,25]
*mytilaspidis*	8 + 5	5	entirely or predominantly black	[1,26]
*proclia*	13 + 6		generally entirely yellow ^(2)^	[1,27,28,29]
unassigned sp.	15	3	entirely or predominantly black	[1,27,28,30,31]
Total sp. ^(3)^	95	28		

Notes: ^(1)^ According to the record by Rosen and DeBach [1] and the authors following them. ^(2)^ Summary comment on the pupal pigmentation of *proclia* group by Rosen and DeBach [1]. ^(3)^ The 27 species in the *vittatus* group of *Aphytis* were transferred to the genus *Paraphytis* [32].

## Data Availability

Data are contained within the article.
